# HBV-IoT: Hierarchical Blockchain-Based Vehicular IoT Network Model for Secured Traffic Monitoring and Control Management

**DOI:** 10.3390/s26082511

**Published:** 2026-04-18

**Authors:** Shuchi Priya, Sushil Kumar, Anjani, Ahmad M. Khasawneh, Omprakash Kaiwartya

**Affiliations:** 1 School of Computer and Systems Sciences, Jawaharlal Nehru University, New Delhi 110067, India; shuchi46_scs@jnu.ac.in (S.P.); skdohare@mail.jnu.ac.in (S.K.) anjani19_scs@jnu.ac.in (A.); 2 Faculty of Information Technology, Philadelphia University, Amman 19392, Jordan; akhasawneh@philadelphia.edu.jo; 3 Department of Computer Science, Nottingham Trent University, Clifton Lane, Nottingham NG11 8NS, UK

**Keywords:** vehicular IoT, hierarchical blockchain, smart contracts, traffic management, edge computing, cybersecurity

## Abstract

Smart vehicles integrated with the Internet of Things (IoT) provide rich data for traffic management, safety, and liability services; however, existing blockchain-enabled vehicular architectures still struggle with consensus scalability, heavy centralized validation, limited interaction-based corroboration, incomplete attack coverage, and rapid ledger growth. In particular, many schemes either optimize single-layer consensus or embed detailed reputation information into every transaction, while pushing most validation to central servers. This leads to bottlenecks under dense traffic and leaves replay, Sybil-assisted 51% attacks on roadside units (RSUs), and man-in-the-middle tampering only partially addressed. In this context, this paper proposes a novel hierarchical blockchain for vehicular IoT (HBV-IoT) model to address the above challenges. An independent transaction for periodic vehicle status reporting and an interaction-based transaction for corroborating data between vehicles in proximity are presented. Three smart contracts are designed to automate the validation and processing of transactions, and to identify compromised or malicious vehicles within the HBV-IoT network. Algorithms for distributed consensus to accept transactions into the blockchain and for vehicle reputation management to enforce edge-level filtering and down-weighting of malicious nodes are implemented. Simulation results demonstrate significant improvements compared to conventional vehicular blockchain approaches, with performance gains validated by 95% confidence intervals. The model supports practical applications, including real-time traffic monitoring, automated e-challan issuance, intelligent insurance claim processing, and blockchain-based vehicle registration.

## 1. Introduction

The Internet of Things (IoT) has emerged as one of the rapidly expanding technologies in the industry, especially in supporting new transportation systems. The number of devices and services connected to the Internet continues to increase, thereby becoming more accessible to end users. The creation of intelligent vehicles is among the most prominent fields of IoT technology usage [1,2]. All companies are integrating IoT technologies in their modern vehicles to gather, process, and distribute information to service providers, traffic authorities, and other vehicles [3]. Such intelligent vehicles can communicate with traffic devices and other vehicles in the area to exchange data, enabling services such as real-time traffic control and optimization, improved driver safety, and collision avoidance. They also assist in automated traffic violation detection and documentation, as well as in insurance claims processing, with accurate liability determination. Nonetheless, connecting cars to networks raises significant concerns, including security, privacy, and scalability.

The majority of smart cars are based on centralized data storage, which introduces security bottlenecks and single points of failure. Centralized systems are prone to data breaches, hacking, and system-wide attacks. In centralized systems, it is hard to ensure the authenticity of data sent by vehicles, especially when different parties may independently require the authenticity of the information [4]. There is a high level of privacy concern due to continuous data collection by vehicle sensors, which include GPS position, speed, acceleration, and driving patterns. The current systems [4,5] face the challenge of balancing transparency and privacy preservation. They are also scalable; as more and more connected vehicles are added, the system scales exponentially, and it cannot effectively support the high volume of transactions and real-time data streams without increasing latency.

The blockchain technology, initially created by Satoshi Nakamoto as the foundation of the Bitcoin cryptocurrency, provides a distributed electronic ledger that avoids reliance on a single centralized authority [6]. It is a distributed digital ledger that removes the reliance on a single centralized authority to store and manage data. Blockchain provides a wide range of features that enhance privacy, reliability, availability, and security for IoT devices and communications. Since its inception, it has mainly been used in digital currency networks, but it has now found multiple applications in other areas as well. Traditional blockchain implementations, such as Proof-of-Work systems, rely on solving computationally hard yet easily verifiable mathematical equations [6,7]. While secure, this approach requires substantial computational resources and energy, making it unsuitable for battery-constrained IoT devices and real-time vehicular applications [8,9].

The authors of [10] propose a Vehicular Network-Based Consensus Algorithm (VBCA) for blockchain-enabled vehicular networks to improve data security and reduce consensus latency. They use a consortium blockchain with roadside units (RSUs) and a Raft-inspired approach, separate transaction and block confirmation to increase decentralization, and rely on smart contracts and leader selection among RSUs. The VBCA suffers in a dense vehicular environment because it relies on a single-layer consensus and requires more communication. Authors designed a blockchain-based data storage architecture for the Internet of Vehicles (BDAV) [11] that explicitly targets latency and query efficiency issues in vehicular data. It proposes delay-aware consensus and data query algorithms that enable blocks to be confirmed and data to be retrieved within strict vehicular delay constraints. It focuses mainly on delay and query efficiency, while storage scalability and long-term ledger growth on RSUs/edge nodes are only partially addressed. The BDAV architecture optimizes queries but may still face state explosion under dense urban traffic. BDAV storage architectures often push more operations to edge/cloud layers, adding extra hops and coordination delays. Dynamic trust management for blockchain-based IoV has recently been proposed in [12]. Authors propose a Dynamic Vehicle Reputation Consensus (DVRC) algorithm that incorporates vehicle reputation into block validation and leader selection to enhance communication reliability and robustness. However, DVRC primarily optimizes the consensus and trust layer of a single blockchain, thereby increasing transaction overhead by adding significant data to each transaction to track reputation scores, historical behavior, and node reliability. DVRC increases total storage overhead and increases ledger size, consuming limited resources on participating nodes in a dense urban environment. The authors of [13] proposed a blockchain-based trust management and social-routing scheme for IoVs that fuses multi-dimensional trust with a lightweight blockchain maintained by RSU to secure multi-hop forwarding.

Existing reputation schemes proposed in [5,6,7,10,11,12] are primarily on updating reputation scores based on single-vehicle messages. A key weakness of these schemes is that they treat each vehicle’s report largely in isolation and rely mostly on how often a single vehicle’s messages match an expected pattern or on coarse crowd feedback, without using strong cross-vehicle corroboration for each specific event. This makes them vulnerable to collusion and coordinated lying, and a small group of malicious vehicles can upvote each other’s false reports and still maintain a high reputation. In HBV-IoT, the proposed UpdateVehicleReputation algorithm computes, for each vehicle, the ratio of high-confidence matched interaction-based transaction records whose reports agree with those of other vehicles observing the same event, and assigns bonuses or penalties accordingly. As a result, a small colluding group can no longer maintain a high reputation merely by upvoting each other’s false reports.

Existing vehicular blockchain methods [6,11,12] do not address effective management of ledger growth. This issue is addressed by [10,14] through middle-level transaction filtering, but still pushes all validated data to a single ledger or attaches heavy reputation metadata to every transaction, which increases the ledger size as vehicle density grows. HBV-IoT addresses this by proposing two independent, interaction-based validation smart contract algorithms that run at RSUs to accept, reject, or locally aggregate events, and forward only compact, consensus-backed results to the global ledger, unlike schemes that push every validated event or reputation update chain-wide.

Early works utilized conventional consensus mechanisms such as Proof-of-Work (PoW), Practical Byzantine Fault Tolerance (PBFT), and Raft-like schemes, which exhibit significant limitations in dense environments, including high energy consumption, low throughput, and long confirmation delays, which are unsuitable for battery-constrained vehicles and latency-sensitive traffic decisions. To address these limitations, the proposed distributed consensus algorithm in HBV-IoT eliminates mining, restricts consensus to RSU validators after local smart-contract filtering, aggregates small batches of pre-validated transactions, and uses reputation-weighted threshold voting with three outcomes (approve, dispute, reject), which jointly reduce communication overhead and latency, and consume low energy while maintaining robustness against malicious or low-reputation validators in dynamic vehicular environments.

In this context, this paper proposes HBV-IoT, a hierarchical blockchain architecture for vehicular IoT networks to restrict all nodes to maintain full blockchain copies and participate in resource-intensive consensus mechanisms. The HBV-IoT is a vehicular network comprising nodes with vastly different computational capabilities. These nodes, known as smart vehicles, have limited computational power, are battery-constrained, and generate multiple transactions per hour. They are traffic devices with medium processing capability, connected to a permanent power supply, and central servers with high computational power. Rather than forcing all nodes to participate equally in consensus mechanisms, HBV-IoT employs a hierarchical approach where smart vehicles periodically generate and store transactions locally, transactions are offloaded to traffic devices when vehicles come within communication range, traffic devices validate transactions and forward them to central servers for final processing, and central servers maintain the global blockchain and provide dispute resolution. The following are the paper’s main contributions.

- A novel four-tier hierarchical blockchain architecture accommodating heterogeneous node capabilities while maintaining security guarantees is presented.
- Independent and interaction-based transaction types are presented to know the state of the vehicles and to enable cross-validation and fraud detection when two vehicles are in proximity to each other.
- Smart contracts are proposed to automate consensus, validation, and dispute resolution mechanisms for vehicle interactions.
- Distributed consensus algorithm and vehicle reputation management algorithm are presented for the traffic device to write transactions on the blockchain, and evaluate the trustworthiness of the vehicles, respectively.
- Security analysis of the HBV-IoT is presented to show its robustness against different types of attacks.
- Simulation results are compared with state-of-the-art approaches to demonstrate the effectiveness of the proposed scheme.

## 2. Related Work

### 2.1. Blockchain in Vehicular Networks

Recent research has explored multiple applications of blockchain technology in vehicular systems. The authors of [9] presented blockchain for autonomous vehicles to address security and privacy concerns through a consortium blockchain approach. Their work emphasizes the importance of decentralized validation for vehicular data security. The authors of [15] presented a detailed review of blockchain-enabled vehicular networks, classifying applications into trust management, privacy preservation, routing, and data sharing, and highlighting scalability and interoperability challenges in practical deployments. The authors of [16] surveyed blockchain-based VANETs, discussing consensus choices, latency constraints, and security requirements specific to highly dynamic vehicular topologies. A more recent overview [10] of blockchain-based applications and architectures for the Internet of Vehicles further categorizes vehicular use cases for secure message dissemination, reputation management, and liability attribution and identifies open issues in cross-domain interoperability. Several works [17,18,19,20] also propose blockchain-inspired lightweight trust or secure routing mechanisms specifically for vehicular networks, where reputation scores and message validity are updated via on-chain records to mitigate the spread of false information and Sybil attacks. These efforts collectively demonstrate that blockchain has matured from conceptual designs toward more specialized vehicular solutions with concrete trust, routing, and data-sharing models.

### 2.2. Hierarchical and Multi-Layer Blockchain Architectures

Hierarchical or multi-layer blockchain architectures have recently gained attention as a way to balance scalability, latency, and trust requirements in IoT and vehicular environments. Authors proposed in [21] a permissioned two-layer blockchain architecture for central bank digital currency. In this architecture, separating interactions between the central bank and commercial banks from those between commercial banks and end-users is an early example of distributing roles and responsibilities across ledger tiers. Similar principles can be applied to vehicular ecosystems by assigning vehicles, roadside units (RSUs), and traffic devices, as well as higher-level authorities, to different blockchain layers. In the vehicular context, authors of [10] introduced a data storage architecture for the Internet of Vehicles where delay-aware consensus and hierarchical query mechanisms effectively decouple local data handling from global ledger maintenance. Authors of [11] employed blockchain for vehicular data security, combining it with tiered storage and processing. A hierarchical blockchain has been proposed as a line of defense against message attacks in VANETs, where lower-layer chains perform localized verification and an upper-layer chain ensures global consistency and auditing [17]. The authors of [18] presented a blockchain-based VANET edge computing-assisted cross-vehicle enterprise authentication architecture (BECA), in which RSUs or edge servers act as local verifiers and miners to reduce latency while maintaining security. The authors of [19] discussed integrating edge computing and blockchain to build safer, more intelligent transportation systems, emphasizing multi-layer trust and task offloading among vehicles, edge nodes, and the cloud. More broadly, recent work [20] on blockchain-based secure vehicular networks demonstrates that multi-tier control and ledger planes can jointly improve scalability and manageability in future C-V2X ecosystems. These hierarchical and edge-assisted designs demonstrate that multi-tier blockchain structures are effective in partitioning responsibilities, reducing on-chain overhead, and improving latency in vehicular environments.

However, existing hierarchical or multi-layer blockchain approaches are typically optimized for specific functions—such as message authentication, enterprise access, or data storage—and do not offer an integrated architecture that simultaneously supports registration, periodic status reporting, cross-vehicle corroboration, malicious-node detection, and application-level services such as traffic monitoring, e-challan, and insurance. In contrast, the proposed HBV-IoT model builds on the layering principles demonstrated by the roles of smart vehicles, traffic devices with edge capabilities, and central authorities within a four-layer hierarchical architecture.

### 2.3. Blockchain-IoT Integration for Diverse Domains

Several authors explored the integration of blockchain with IoT across multiple sectors, providing insights applicable to vehicular networks. The authors of [22] developed a General Data Protection Regulation-compliant architecture for sharing personally identifiable information and protected health information using blockchain. Their approach of separating confidential and non-confidential data storage with smart contract-mediated access applies to vehicular networks. The authors of [23] proposed Blockchain-Enabled Secure and Trusted Public Emergency Services that integrate home-based IoT infrastructure with blockchain for efficient emergency detection and response. Their use of access control lists maintained in IoT controllers provides a model for vehicular network security. The authors of [21] presented a self-sovereign identity management system for medical IoT to demonstrate decentralized identity verification without dependency on a central authority. This approach is adaptable for vehicle registration and licensing. This multi-level architecture provides insights for hierarchical vehicular networks. The authors of [24] developed an IoT-enabled blockchain network for agricultural insurance to optimize processing, security, and settlement. Their transaction validation approach, which uses multiple corroborating sources, is analogous to interaction-based transactions in vehicular networks.

### 2.4. Blockchain Security and IoT Vehicular Foundations

The authors of [8] analyzed shortcomings of cyber–physical systems used with IoT. They identified how blockchain can mitigate vulnerabilities through cryptographic security, non-repudiation, and immutability, thereby meeting foundational security requirements for vehicular networks. The authors of [25] discussed enhanced IoT ecosystem security with blockchain, and ref. [26] focused on protecting IoT device data using distributed validation and cryptography. Recent vehicular security work includes false message detection in IoV using machine learning and vehicle consensus, heterogeneous broadcast and key-insulated multi-receiver signcryption schemes for IoV, and trust-aware sensing and positioning mechanisms in vehicular systems. These highlight that future transportation systems require robust, cryptography-backed trust infrastructures. The authors of [27] propose a hybrid incentive-and-reputation mechanism to improve data quality and participation in vehicular crowdsensing while controlling costs. The reputation and reward models depend on accurate evaluation of data quality, which itself can be noisy or subject to attack. Further, blockchain-driven trust management and routing schemes (BTMRs) [14] in IoV employ consortium or permissioned blockchains to maintain social-routing metrics and reputations, combining on-chain reputation with lightweight consensus to enhance security and energy-efficient communication in highly dynamic vehicular environments. Other research on future transportation systems focuses on the Internet of Vehicles and the advancement of electric vehicles [28,29,30].

### 2.5. Positioning of the Proposed HBV-IoT Model

Overall, the above-mentioned studies mainly focus on specific aspects improved through blockchain and IoT—such as energy trading, emergency services, identity management, data storage, trust, routing, or authentication—and often address a single application or function at a time. A tabular-based comparison of HBV-IoT with the five most relevant state-of-the-art blockchain-enabled vehicular and IoV architectures, highlighting where our design converges with or departs from existing solutions, is given in Table 1.

## 3. Propose HBV-IoT Model

This section first presents the system architecture of the HBV-IoT model. Second, the smart contracts to automate the validation and processing of transactions within the HBV-IoT network are presented. Third, the lightweight and distributed consensus and vehicle reputation management algorithms of HBV-IoT are presented.

### 3.1. System Architecture

**Figure 1:**
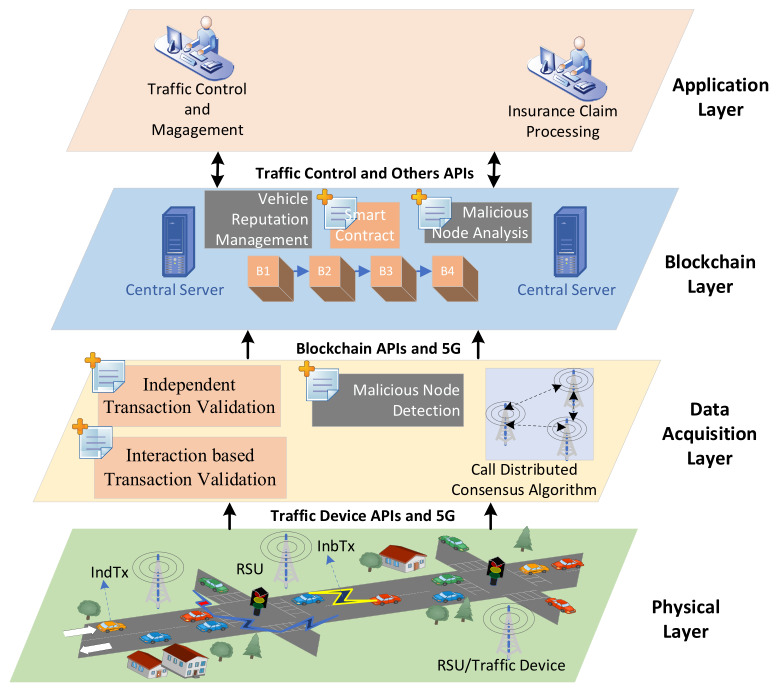
Layered architecture of HBV-IoT.

The HBV-IoT model consists of a four-layer architecture: physical, data acquisition, blockchain, and application layers, each with a distinct role in the vehicular IoT ecosystem as shown in Figure 1. The physical layer hosts smart vehicles and traffic devices (RSUs) such as speed cameras, traffic lights, toll booths, parking systems, and highway monitoring stations, each equipped with processing and communication units to interact with regional and government servers for vehicle and traffic monitoring. Smart vehicles act as primary data sources and run a Vehicle Monitoring Interface (VMI) to continuously collect sensor data (location, speed, acceleration, events, etc.). The VMI constructs independent transactions (IndTx) for periodic status and interaction-based transactions (InbTx) when nearby vehicles exchange corroborating state information. It signs each transaction with the vehicle’s private key, and buffers them locally without executing smart contracts to save onboard resources. Local storage (e.g., HDD or SD card) is used to temporarily store independent and interaction-based transactions before they are offloaded to RSUs. Local storage acts as a short-term, privacy-preserving cache for these signed transactions until connectivity is available. When the vehicle enters the range of an RSU over Bluetooth, Wi-Fi, or 5G, the VMI offloads the buffered transactions to the nearest traffic device for further validation and processing.

The data acquisition layer begins operating when transactions reach the RSUs. Each traffic device exposes APIs that receive the local blockchain segments and transaction pools from nearby vehicles’ VMIs and translate them into the format required by the global blockchain. Before processing individual transactions, the RSU consults the global ledger to retrieve the current reputation and state of each vehicle—MONITOR, ISOLATE, or PENALIZE—states that are periodically computed by the malicious node-detection contract at the central servers. When a vehicle is in the PENALIZE state, the RSU drops all transactions, captures the event, and does not send them to the next destination. With vehicles in MONITOR or ISOLATE states, the RSU is more conservative with them, such as by giving them lower weights of trust or marking their data to undergo extra validation.

The validation of transactions in the RSU is performed with the help of two smart contracts: one of them is used to verify independent transactions with the integrity, signatures, and vehicle reputations, and another is used to verify interaction-based transactions by finding the counterpart records of two vehicles and identifying inconsistencies as suspicious or malicious. Approved batches are added to the local chain of the RSU, and Disputed and Rejected batches are then forwarded to the central servers, where validated transactions are pooled on each RSU. A distributed consensus algorithm is executed on the pool to classify batches into Approved, Disputed, and Rejected subsets. Central servers aggregate blocks from RSUs, finalize them into the global blockchain, and log validation outcomes, disputes, and rejections per vehicle. Some of these are input to the update-reputation-management algorithm at RSU, enabling the system to enforce edge-level transaction filtering and isolate vehicles that continually produce anomalous or conflicting blocks.

**Figure 2:**
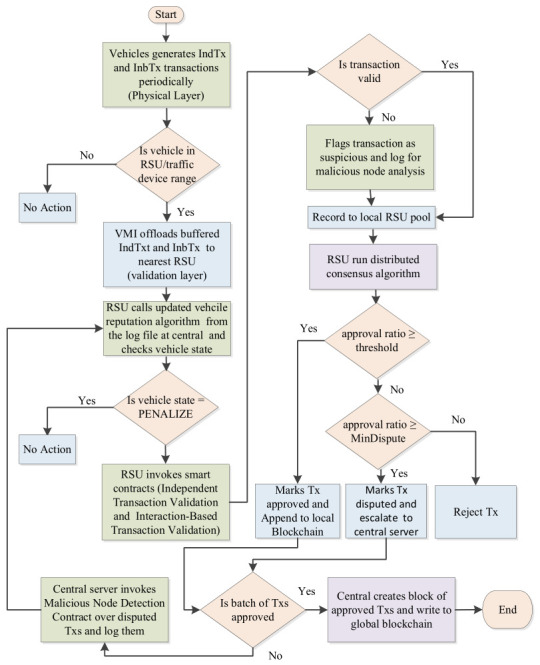
Logic flow of HBV-IoT model.

The blockchain layer will not only maintain the global ledger but also execute Smart Contract 3 (malicious-node detection). Central servers call upon this contract on a per-batch basis for all transactions and recorded anomalies to compute new reputation scores and the categorical states (MONITOR, ISOLATE, PENALIZE) for all vehicles. The resulting states are stored on the global blockchain and exposed via APIs so that RSUs can fetch and apply them in near real time. As a result, edge-level filtering is continuously informed by global behavior: RSUs can drop or down-weight transactions from penalized vehicles, prioritize traffic from trustworthy nodes, and contribute fresh evidence back to the central layer, closing the loop between local observation and global enforcement. Finally, the application layer consists of traffic monitoring, e-challan issuance, insurance claim processing, and vehicle registration applications that use global blockchain data via traffic control and other APIs for various purposes. To understand the overall logic of the system, we presented a flowchart in Figure 2. We also present a concise working logic of the system step-wise as follows:

1. Vehicle generates data
  - VMI continuously collects sensor data.
  - VMI periodically creates:
    ∘ IndTx for periodic status.
    ∘ InbTx for exchange corroborating data.
  - Each Tx is signed with the vehicle’s private key.
  - Tx is buffered locally until no RSU is in its proximity.
2. Vehicle enters RSU range
  - VMI offloads buffered IndTx and InbTx to the nearest RSU.
3. RSU checks vehicle state
  - The RSU queries the global blockchain for the vehicle’s current reputation and state: MONITOR, ISOLATE, or PENALIZE, which are set by the malicious-node detection contract at the central layer.
    ∘ If the state is PENALIZE, RSU drops all transactions and the log event.
    ∘ Else, the state is MONITOR or ISOLATE, the RSU continues with validation, but may treat the vehicle more cautiously.
4. RSU invokes Smart Contract 1 (Independent Transaction Validation)
  For each IndTx:
  - Call ValidateIndependentTransaction(IndTx).
  - If valid, add to local RSU pool.
  - Else, mark as rejected and log for central analysis.
5. RSU invokes Smart Contract 2 (Interaction-Based Transaction Validation)
  For each InbTx pair from two vehicles:
  - Call ValidateInteractionBasedTransaction(txA, txB).
  - If approved, add canonical interaction record to local RSU pool.
  - If approved with low confidence, it adds and flags it as suspicious.
  - If rejected/flagged, store for malicious-node analysis.
6. RSU: run distributed consensus
  RSU validators execute DistributedConsensus(TxBatch, validators) over the validated transactions pool.
  - Validators (RSUs) vote Approved, Disputed, or Rejected.
  - Append Approved to RSU’s local chain; forward Approved and Disputed to central servers.
7. Central servers: commit and log
  Central servers aggregate blocks from RSUs, finalize them into the global blockchain, and log validation outcomes, disputes, and rejections per vehicle.
8. Central servers invoke Smart Contract 3 (malicious-node detection)
  Periodically call DetectMaliciousNodes(BatchOfTransactions) to identify compromised or malicious vehicles.
9. RSUs receive updated states
  RSUs regularly call the updated vehicle reputation algorithm, retrieve the state (MONITOR/ISOLATE/PENALIZE) from the central server, and apply it to steps 3–6, enforcing edge-level filtering and down-weighting for suspicious nodes.
10. End of cycle; repeat
  Vehicles continue generating transactions; the loop repeats with updated reputations and states.

### 3.2. Transaction Types and Structure

The HBV-IoT model supports two primary transaction types, each serving distinct purposes.

1. Independent Transactions (IndTx)—Independent transactions represent vehicle state at specific time intervals, which consist of the following structure: Header: {TID, PTID, IK, TS, SIGN} and Payload: {LOC, SP, ACC, DIR, TEMP, HU, BET, EVT, CHECK}. They contain a header which includes transaction ID (TID) denoting a unique 256-bit hash identifying the transaction, previous transaction ID (PTID) denoting the hash of the previous transaction, Identification Key (IK) representing the vehicle’s unique identifier in the network, timestamp (TS) representing the precise time of transaction creation, and signature (SIGN) representing the elliptic curve digital signature algorithm (ECDSA) signature using the vehicle’s private key. All are part of the header fields along with the data generated during the given timeframe. These types of transactions are generated by the VMI frequently at a periodic interval to store device related information such as location (LOC): GPS coordinates {latitude, longitude, altitude}; speed (SP); acceleration rate (ACC) of velocity change (m/s^2^); direction (DIR): heading angle (0–360 degrees); environmental data such as temperature (TEMP), humidity (HU), and weather conditions; vehicle status like battery level (BET), sensor health (SHE), system diagnostics, and events (EVT)—hard braking, collision warnings, and maintenance alerts; and checksum (CHECK) using CRC32 for data integrity verification, into the local blockchain network. These transactions are stored locally on the node until they come within range of a traffic device, which can offload them to the global blockchain network for transfer.
2. Interaction-Based Transactions (InbTx): These transactions are event-based when two smart vehicle nodes come within range of each other. They enable cross-validation and fraud detection. This transaction is similar to an independent transaction, but it will also contain the signature of the other nodes participating in the interaction. The structure of InbTx can be given as Header: {TID, IK, ${IK}_{1}$, ${IK}_{2}$, SIGN, TS}; Dual Signatures: {${SIGN}_{1}$, ${SIGN}_{2}$, ${HASH}_{1}$, ${HASH}_{2}$; and Shared Data: {*REL_P*, *REL_V*, INT_D, *MUT_A*}. The signature of this block will have TID, Identification Key of the current vehicle (${IK}_{1}$), Identification Key of the second vehicle (${IK}_{2}$), Hashed Signature of data of the current vehicle (${SIGN}_{1}$) and Hashed Signature of data of the second vehicle (${SIGN}_{2}$) and timestamp (*TS*) representing the time of interaction detection. It also contains the Merkle hash of vehicle 1’s data (${HASH}_{1}$) and Merkle hash of vehicle 2’s data (${HASH}_{2}$). The shared data between vehicles includes relative position (REL_P), relative velocity (REL_V), the duration of vehicles remaining in range (INT_D), and mutual awareness (MUT_A) to confirm that both vehicles detected the interaction. This transaction is generated in both smart vehicles and stored locally until they come within range of a traffic device, at which point it is offloaded to the global blockchain network. As the transaction is generated in both smart vehicles before being saved to the global network, validation is required to ensure the transactional data is identical across both nodes; only the data blocks that are consistent across both devices will be saved. In the validation mechanism, when vehicles generate interaction-based transactions, both transactions must contain identical data for cross-vehicle fields. Inconsistencies indicate tampering or compromised nodes and trigger an investigation. This introduces an additional level of authenticity and non-repudiation in the network, as any malicious user cannot make any malicious entry into the network, as it must be verified by data blocks generated by interacting vehicles.

### 3.3. Threat Model

The proposed HBV-IoT considered five types of attacks during the process of generating vehicle transactions for the global blockchain update. These are explained as follows.

1. Double-Spending Attack: A malicious vehicle tries to use the same funds or credit twice by creating two different transactions that consume the same balance, often with identical or near-identical timestamps, so they appear concurrent. The goal is to have both transactions accepted before the system notices the conflict, effectively “spending” more than the true balance.
2. Sybil Attack: An adversary creates numerous fake identities of the vehicles (several public keys/IDs) and introduces them into the system in such a way that one attacker can be represented as a great number of independent nodes. In so doing, the attacker tries to sway consensus votes, reputation scores, or validation results by acting upon a large portion of the apparent participants, although they are controlled by a single party.
3. 51% Consensus Attack: The attacker targets validators (traffic devices/RSUs). When the attacker can breach or manage a majority of the validator power (e.g., over 50% of the RSUs or the effective reputation weight thereof), they will be able to collaborate to endorse fraudulent transactions, censor legitimate ones, or rewrite recent sections of the ledger. This is a collusion attack on the consensus process at an infrastructure level.
4. Transaction Replay Attack: Attacker captures a legitimate transaction and replicates it several times. The attacker logs an authentic exchange because it passes through the network and subsequently repeats it (replays) several times without the awareness of the vehicle. Unless the system properly imposes the uniqueness of transaction IDs, timestamps, or sequence numbers, the same action (e.g., a payment, status report, or authorization) might be performed multiple times.
5. Man-in-the-Middle (MITM) Attack: In a MITM attack, the adversary positions themselves between the vehicle and the traffic device/RSU, intercepting and possibly altering messages in transit. They might change sensor readings, locations, or control commands while still trying to pass signature or protocol checks, aiming to inject false data or hide true events without endpoints immediately realizing that messages have been tampered with.

### 3.4. Blockchain Smart Contracts and Algorithms

This section explains smart contracts and algorithms used to validate transactions, update the reputation, and detect malicious vehicles.

A. Smart Contracts

Smart contracts automate the validation and processing of transactions within the HBV-IoT network, and are presented in this section. Three smart contracts, Independent Transaction Validation, interaction-based transaction validation, and malicious-node detection, are designed. Traffic devices and central servers call these contracts.

1. Smart Contract 1: Independent Transaction Validation

This contract provides confirmation of independent transactions between vehicles. This contract verifies single-vehicle (independent) transactions (periodic status updates (SP, LOC, ACC)) prior to their acceptance. It checks the digital signature, the time sequence, sensor plausibility, and checksum to confirm that the information is original, timely, and undistorted. Once all validations are complete, the transaction is added to the local blockchain, the global blockchain, or both. Vehicles with low reputation can be marked as needing additional screening; that is, they are not rejected (c.f. Algorithm 1). It mainly verifies the signature and identity by calling the Verify_Signature (Ver_S) function. It verifies the digital signature of the header with the registered public key of the vehicle. In case of failure of the signature, the transaction is rejected because it is not for that vehicle. This guarantees authenticity and non-repudiation so that the only valid signature of the Identification Key (SIGN, IK) that can be produced by that vehicle is the signature of its private key. It performs temporal consistency checking by calling the Check_Temporal_Consistency (Check_TC) function. It guarantees that the timestamp falls within a small window (e.g., ±5 s) of the node’s current time to avoid replay or delayed injection. It queries the previous (SIGN, IK) of the same vehicle and ensures that the new timestamp is strictly greater. It does not tolerate non-monotonic or stale timestamps, which exclude re-ordering and time-based attacks. The sensor data is validated by the contract, referring to the validate_Sensor_Data(Val_SD) function. It verifies SP and ACC of the vehicle in the valid range, and SP in the geographical network area of configuration. If any transactions involve physically impossible values, such as negative speed or teleportation, they are rejected as likely forged or faulty. The data integrity is verified by calling the Verify_Data_Integrity (Ver_DI) function. It recomputes a CRC32 (or similar) checksum over the payload and compares it to the stored checksum. If any mismatch is found, it indicates corruption or tampering in transit and storage, leading to rejection. The reputation of each vehicle is flagged by calling the reputation_Check function (Rep_C). It reads the vehicle’s current reputation score (0–100). Transactions from low-reputation vehicles (e.g., <30) can be marked “validated but suspicious,” so higher layers (e.g., a malicious-node detector) watch them more closely, even if this single transaction passes all checks. Only if all checks pass, the transaction is appended to the local blockchain, with proper linkage through TID/PTID by calling the Store_In_Local_Blockchain (Store_LB) function.
**Algorithm 1.** Contract ValidateIndependentTransaction(transaction)**Input**: transaction **Output**: ValidationResult {isValid: Boolean, reason: String} **Begin**
1. **Function** *Ver_S()* {
           Use the vehicle’s public key to verify the ECDSA signature.
           **If** ( signature invalid) {
                    **Return** {isValid: false, reason: “Invalid signature”}
           } **End** if
    }**End** function
2. **Function** *Check_TC*() {
           Verify timestamp is within ±5 s of the current time.
           Confirm the timestamp is after the previous transaction’s timestamp.
           **If** ( inconsistent) {
               **Return** {isValid: false, reason: “Invalid timestamp”}
             } **End** if
        }**End** function
3. **Function** *Val_SD*() {
           Check SP is within the vehicle’s physical capabilities (0–250 km/h)
           Verify ACC is within realistic bounds (−10 to +10 m/s^2^)
           Confirm that the GPS coordinates are within the network bounds.
           **If** (any violation) {
               **Return** {isValid: false, reason: “Implausible sensor data”}
           } **End** if
        }**End** function
4. **Function** *Check_PT*() {
          Retrieve PTID
          Verify it exists in the local or global blockchain.
          Confirm chronological order
          **If** (failed ) {
                **Return** {isValid: false, reason: “Previous transaction not found”}
            } **End** if
        }**End** function
5. **Function** *Ver_DI*() {
          Recalculate CRC32 checksum from payload
          Compare with the provided checksum.
          **If** (mismatch ) {
                  **Return** {isValid: false, reason: “Data corruption detected”}
          } **End** if
        }**End** function
 
6. **Function** *Rep_C*() {
          Retrieve the vehicle’s current reputation score.
          **If** ( score < 30: Flag for manual review (suspicious vehicle)
                   Continue processing, but mark with lower confidence
            } **End** if
        }**End** function
 
7. **Function** *Store_ LB*()  {
            Add a validated transaction to the local blockchain.
            Update transaction index for quick retrieval
            **If** (all checks pass) {
                  **Return** {isValid: true, reason: “Transaction validated”}
            } **End** if
        }**End** function
 
**End**

2. Smart Contract 2: Interaction-Based Transaction Validation

This smart contract verifies the authenticity of transactions txA and txB generated by two vehicles, say A and B, when they interact (c.f. Algorithm 2). The contract verifies both vehicles’ signatures (SIGN_1_, SIGN_2_) by invoking the Initial_Signature_Verification (Int_SV) function. If either signature fails, both transactions are rejected for that interaction, since the cryptographic origin cannot be trusted. The function Cross_Vehicle_Consistency_Check (Cros_VCC) compares shared fields such as REL_P, REL_P, DUR, and interaction type within the ACC limit to compute a match score as

SCORE=MatchingFields TotalFields×100

This provides a continuous measure of how closely the two vehicles agree. The function Data_Plausibility_Check (Data_PC) enforces physical constraints on the interaction itself. It checks if the relative velocity does not exceed a configured limit (e.g., 150 km/h), the duration is not unrealistically short (e.g., <2 s) for a meaningful interaction, and the relative position does not exceed the typical communication range (e.g., 500 m). In normal urban and highway conditions, the relative velocity between two interacting vehicles can hardly be greater than 150 km/h when both are on the same general traffic stream. Such an upper-bound filter can filter out physically implausible interactions due to spoofed GPS or injected data. It thus acts as a sanity check, and any alleged interaction with a relative at a speed greater than this point would probably be erroneous or malicious and can be discredited or marked. A 2 s interaction window is sufficiently large to achieve a stable approach, yet small enough to be friendly in traffic jams. This minimizes the chances that transient or chance radio contacts are registered as valid, corroborated transactions. The requirement that the relative position difference be above 500 m along a given stretch of road ensures that only interactions that are spatially separated enough are counted when applying certain validation rules. Practically, this limit can be reasoned as the distance further than normal GPS jitter and short-range lane variations; hence, it grabs out interactions wherein position noise may prevail. Combined with REL_V and DUR, it is used to separate meaningful interactions, position noise artifacts, and enhance the validity of interaction-based validation. The Timestamp_Synchronization (Time_S) operation determines the difference between the timestamps of the two transactions, txA and txB, to be within the tolerance threshold; otherwise, both transactions are meant to signal a clock tripping, spoofed data, or delayed transmission that must be rejected or re-examined by the central server. Merkleroots are recalculated on the data of every vehicle and compared to Hash 1 and Hash 2 on the transactions with the help of the Merkle_ Hash Verification (Mer_HV) functionality. In case of any mismatch, it signifies that the data in the transaction has been modified after signing and is considered tampering. The Consensus_Decision (Con_D) function decides to approve the transactions txA and txB. t checks on the match score. With high scores on the match, transactions are granted immediately, and the vehicles obtain small reputation rewards through the Reputation_Update (Rep_U) command. Borderline transaction matches are also held but flagged. In case the score is low, it results in loss of reputation for both vehicles, signifying non-repudiation and fraud detection. The score in ConD ( ) is a brief, numerical index of the degree of agreement between two interaction-based transactions on all fields of agreement. The computation of a normalized percentage of matched fields and comparison against the three bands (high, medium, low) allows the smart contract to approve interactions that are clearly consistent, accept interactions that are borderline, and reject highly inconsistent interactions. This causes fewer false positives due to minor sensor or timing noise and still lets HBV-IoT punish repeated discrepancies as evidence of malicious intent.
**Algorithm 2.** Contract ValidateInteractionBasedTransaction(transaction1, transaction2)
        **Input**: txA, txB 
        **Output**: ValidationResult {isValid: Boolean, matchScore: Float, action: String}
        **Begin**
        1. **Function** *Int_SV* () {
                    Verify txA signature using Vehicle A’s public key
                    Verify txB signature using Vehicle B’s public key
                    **If** (either fails) {                              **Return** {isValid: false, action: “Reject both transactions”}
                 } **End** if
        } **End** function
        2. **Function** *Cros_VCC* () {
                    Extract shared data fields from both transactions {REL_P, REL_V, DUR,  InterationType }
                    Compare values with the tolerance threshold.
                    Calculate consistency *SCORE* 
                    **If** (Score < 80%) {                              Inconsistency detected between vehicles.                              Mark Vehicle with inconsistency count++
                      } **End** if
                }**End** function
        3. **Function** *Data_PC* () {
                    **If** (REL_V> 150 km/h ) {  Flag as unlikely  } **End** if
                    **If** ( DUR < 2 seconds ) { Flag as too brief for interaction  } **End** if
                    **If** ( REL_P > 500 m ) { Flag as exceeding range } **End** if
                  Check against recent independent transactions
                  Verify LOC consistency 
                } **End** function
        4. **Function** *Time_S* () {
                 **If** ( |timestamp1 − timestamp2| > tolerance ) {                       **Return** {isValid: false, action: “Timestamp mismatch”}
                   } **End** if
                }**End** function
        5. **Function** *Mer_HV* () {
                    Regenerate Merkle trees from vehicle data.
                    Compare calculated hashes with provided hashes (HASH_1_, HASH_2_)
                    **If** (mismatch ) {                           Possible data tampering detected                           Flag the vehicle for investigation
                    } **End** if
                }**End** function
        6. **Function** *Con_D*() {
                   **If** (Score >= 95%) {                           **Return** {isValid: true, action: “Approve both transactions”, matchScore: Score}
                     } **End** if
              **If** (80%<=Score<95%) {
                      **Return** {isValid: true, action: “Approve with lower confidence”, matchScore: Score}
                     } **End** if
              **If** (Score<80%) {                       **Return** {isValid: false, action: “Flag for central authority review”}
                     } **End** if
                }**End** function
        **7. Function** *Rep_U*() {
                  **For** valid transactions                          Vehicle_A.reputation += 1                          Vehicle_B.reputation += 1
                **End** for
                **For** invalid transactions                        Vehicle_A.reputation −= 10                        Vehicle_B.reputation −= 10
               **End** for
               }**End** function
        **End**

3. Smart Contract 3: Malicious-Node Detection

This smart contract periodically scans recent transactions and interaction outcomes to detect patterns of misbehavior and identify compromised or malicious vehicles. It aggregates per-vehicle inconsistency rates, physical anomalies such as impossible SPs and LoCs, and location-spoofing indicators to compute a maliciousness score by invoking the Analyze_Inconsistency_Patterns (Ana_IP) function. The inconsistency rates (Ins_Rate) and the physical anomalies rate (Ana_Rate) are computed as

Ins_Rate=inconsistent_interactions/total interactions

Ana_Rate=physically_impossible_events/total_events

Based on this score, it decides whether to monitor, temporarily isolate from consensus, or penalize and fully isolate a vehicle, while notifying authorities and updating its on-chain reputation. The Location_Spoofing_Detection (Loc_SD) function checks whether a vehicle appears in two locations that would require impossible speeds or overlapping presence in incompatible lanes. Each confirmed spoofing event contributes to a location suspicion component. The Reputation_Scoring (Rep_S) function provides a single scalar that can drive policy thresholds that determine whether a vehicle should be in MONITOR, ISOLATE, or PENALIZE states. The assignment of these terms different weights α, β, and γ recognizes that not all anomalies are equally predictive. Inconsistent interactions (InconsistencyRate) may be minor compared to verified physical anomalies (AnomalyRate), but location spoofing proven (LocationSuspicion) is usually a good indicator of compromise. A single normalized score is compared to thresholds (score < 0.3 should be in MONITOR, score 0.3–0.7 ought to be in ISOLATE, and score > 0.7 ought to be in PENALIZE) (c.f. Algorithm 3). This makes policy decisions simple. Minor and unpredictable problems keep a vehicle under surveillance, medium problems result in a temporary lack of consensus, and high metrics are motivated by continuous discrepancies or hard data of spoofing, which initiates severe punishments and network-wide bans. A scalar score with weighted elements and tiered penalties allows HBV-IoT to react very harshly to vehicles with genuinely malicious intent, but not yet subject to penalties, to honest vehicles with only occasional low-severity anomalies. The function Take_Action (Take_A) is called when a vehicle is in either state MONITOR, ISOLATE, or PENALIZE, and takes action based on the score.
**Algorithm 3.** Contract DetectMaliciousNodes()
        **Input**: Batch of Transactions
        **Output**: {SuspiciousVehicles: List, Actions: List}
        **Begin**
        1. **Function** *Ana_IP*() {                             **For** each vehicle V                               InCount[V] = Count of inconsistent interaction transactions                               Ins_Rate[V] = InCount[V]/TotalInteractions[V]                               **If** ( In_Rate[V] > 0.15 )  {                                        Mark V as potentially malicious                                } **End** if                      **End** for
                 }**End** function
        2. **Function** *Loc_SD*() {                           Compare GPS coordinates with cellular tower data                    **If** (vehicle reported at multiple locations simultaneously) {                             Flag as location spoofing                      } **End** if
                }**End** function
        3. **Function** *Rep_S*() {                             Calculate Maliciousness Score
                     Score = (InconsistencyRate × α)+ (AnomalyRate × β)+
                     (LocationSuspicion × γ)
                      where  α + β + γ = 1                         **If** (Score < 0.3) {                                   MONITOR by increasing check frequency                       } **elseif**  (0.3 <= Score < 0.7) {                                ISOLATE the vehicle for some specified time
                     } **else** {                                PENALIZE, that is long-term isolation and report to authorities                      } **End** if
        } **End** function
        4. **Function** *Take_A*() {
                 **If** (MONITOR) {                        Log increased surveillance
                    } **End** if
                      **If** (ISOLATE) {                             Revoke the vehicle’s consensus participation rights                                 Prevent acceptance of transactions from this vehicle                                 Set expiration: 24 hours                              } **End** if                          **If** (PENALIZE) {                                Broadcast a malicious node alert to all traffic devices                                 Prevent any transaction acceptance                                 Notify RTO and government authorities                                 Trigger insurance policy review                               }**End** if                            }**End** function
        **End**

B. Algorithms

(1) Distributed Consensus Algorithm

The proposed distributed consensus algorithm (c.f. Algorithm 4) for HBV-IoT is a reputation-weighted voting process implemented in RSU/traffic-device validators. It decides which transactions are accepted into the blockchain. All the transactions generated by smart vehicles are grouped into batches to reduce overhead. Each batch is sent to all available validators (RSUs) for checking. Every validator has a reputation score. It is normalized into a weight, so high-reputation RSUs influence the decision more than low-reputation ones.

Voting to approve the transactions is performed as follows. For each transaction, validators respond with APPROVE or REJECT. The algorithm sums the weights of approving validators and divides by the total weight of the responding validators. It gives an approval ratio in [0, 1]. The decision rules for approving the transactions are formulated as follows. If the approval ratio is ≥70%, then the transaction is approved and appended to the local blockchain. If 40%≤ of the approval ratio is <70%, then the transaction is marked as disputed and escalated to layer-3 central servers for further analysis. If the approval ratio is <40%, then the transaction is rejected and not stored in the blockchain. If no or very few validators respond within a timeout, then the transaction is treated as disputed and deferred to the central authority for further processing. The algorithm produces three output sets (Approved, Rejected, Disputed) along with the total consensus time. It then forwards Approved/Disputed to layer-3 for final recording or analysis in the global blockchain. The 70% approval threshold requires a supermajority of weighted trust to accept a transaction. In classical Byzantine fault-tolerant (BFT) protocols, safety is often guaranteed as long as the fraction of malicious participants is less than 1/3, leading to 2/3-type thresholds for commits. In HBV-IoT, weights are not uniform but reflect reputation and require at least 70% of the total weight to approve and accept a transaction only when the overwhelming majority of trusted capacity agrees under the assumption that the total reputation of malicious validators is significantly smaller than that of honest validators, which is enforced by the reputation system. This threshold ensures that malicious validators alone cannot routinely push bad transactions through because they would need to control most of the weighted trust. Below about 40% weighted support, there is clear disagreement, so the safest action is to reject the batch outright. The intermediate band 0.40 ≤ ratio < 0.70 is treated as uncertain rather than forced into acceptance or rejection. In this range, there is a significant minority of weight that disagrees with the majority. This may be due to some RSUs seeing the event and coordinating, but not yet dominant, malicious behavior. Automatically committing or discarding such transactions would either risk accepting controversial or adversarial data or partially observed events. Escalating them as “disputed” to the central layer allows more global checks to be applied.
**Algorithm 4.** DistributedConsensus(TxBatch, Validators)**Input:**
    TxBatch    // list of candidate transactions tx
    Validators // RSUs with reputation scores
**Output:** Approved, Rejected, Disputed
**Begin**
Approved = 0 ; Rejected = 0 ; Disputed = 0
  // Normalize reputations to weights
  totalRep = Σ v.reputation  for v in Validators
  **for** each v in Validators
       v.weight = v.reputation / totalRep
**end** for
Threshold  = 0.70   // approve if ≥ 70% weighted support
MinDispute = 0.40   // dispute if in [40%, 70%)
**for** each tx in TxBatch
         // send tx to all validators and collect votes
         Responses = *getVotesFromValidators*(tx, Validators, timeout)
         weightedApprove = 0
           totalWeight  = 0
         **for** each (v, vote) in Responses
                  totalWeight = totalWeight + v.weight
         **end** for
         **if** (vote == APPROVE)
                 weightedApprove = weightedApprove + v.weight
           **end** if
         **if** (totalWeight == 0)
                    Disputed = Disputed ∪ {tx}                         continue
           **end if**
                      ratio = weightedApprove / totalWeight
         **if** ( ratio ≥ Threshold) {
                 Approved = Approved ∪ {tx}
                 *appendToLocalBlockchain*(tx)
           } **elseif** ( ratio ≥ MinDispute)  {
                 Disputed = Disputed ∪ {tx}
                 *escalateToCentralAuthority*(tx)
          } **else** {
                 Rejected = Rejected ∪ {tx}
           } **end** if
         **end** for
**return** (Approved, Rejected, Disputed)
**End**

(2) Vehicle Reputation Management

The UpdateVehicleReputation algorithm periodically recalculates each vehicle’s trust score based on its recent behavior, then stores the new score on-chain. It first checks recent transactions over the past 100 days; if most are approved and few are rejected/disputed, the vehicle receives a positive consistency bonus (*ConsBonus*). If the majority of bad or disputed transactions are detected, they cause a consistency penalty (*ConsPenalty*). Then it evaluates interaction quality. If the vehicle’s interaction-based transactions frequently match other vehicles’ data with high confidence, its high matching rate gives an interaction bonus (IntBonus); otherwise, frequent mismatches decrease it and give an interaction penalty (*IntPenalty*). Next, it examines compliance with traffic rules (violations and speeding events). If there are no violations, vehicles earn a compliance bonus (CompBonus); each violation and speeding event adds to a compliance penalty (*CompPenalty*) that reduces reputation. It also considers inactivity as follows. A vehicle does not remain active for very long (e.g., 30 days), slowly reducing the score by applying an inactivity penalty (*InactPenalty*) to avoid indefinitely trusting inactive nodes. All bonuses and penalties are added to the current score, clamped to the range of 0–100, and the updated reputation, along with context (rates, violations, inactivity), is written back to the blockchain as the new trust state for that vehicle. The selected thresholds in Algorithm 5 are explained by the way they will form the trade-off between swiftly isolating malicious vehicles and preventing innocent ones from being unfairly penalized. The consistency levels on recent transactions, i.e., ConsBonus at 0.80 or 0.95 and ConsPenalty less than 0.50, make sure that only vehicles that show significantly strong behavioral patterns get high levels of positive reputation promoted. Conversely, continually bad behavior is followed by severe punishments. Likewise, the interaction-quality thresholds (IntBonus) for match rates over 0.90 and IntPenalty under 0.70 were chosen to reward those vehicles whose interaction-based transactions are always similar to those with high confidence and penalize vehicles that often disagree with neighbors. These cut-offs ensure a high detection rate but are tolerant of sporadic false matches caused by sensor noise or rare collisions. A high reward on the days of full compliance with no infractions and no speeding, and a proportional penalty to the number of violations and speeding cases, are also a sure way to make sure that repeated misbehavior of traffic conduct will soon push the reputation to the negative, despite the apparent local consistency of transactions and interactions. The inactivity penalty, which activates after 30 days with a floor-based increase, prevents long-unused vehicles from being permanently trusted.
**Algorithm 5.** UpdateVehicleReputation(vehicleID)**Input**: vehicleID **Output**:  NewRep  // in [0, 100] **Begin**
  CurrentRep = getReputation(vehicleID)
  RecentTx   = getLastNTransactions(vehicleID, N = 100)
 
**if** ( size(RecentTx) > 0 )  {
        valid   = count(tx.status == "APPROVED")
        rate   = valid / size(RecentTx)
        **if** ( rate > 0.95 ) {  ConsBonus = +5  }
        **elseif** (rate > 0.80) {  ConsBonus = +2 }
        **elseif** ( rate < 0.50 ) { ConsPenalty = −15 } 
        **end** if
}**else {**
        ConsBonus = 0 ; ConsPenalty = 0
} **end** if
 
InterTx  = getInteractionTransactions(vehicleID)
**if** ( size(InterTx) > 0 ) {
        matched = count(itx.matchStatus == "MATCHED_HIGH_CONF")
        mRate  = matched / size(InterTx)
        **if** ( mRate > 0.90 ) { IntBonus = +3 }
        **elseif** ( mRate < 0.70 ) {  IntPenalty = −8 
        } **end** if
        **else** {
                  IntBonus = 0 ; IntPenalty = 0
} **end** if
 
Viol   = countTrafficViolations(vehicleID)
Speed  = countSpeedingIncidents(vehicleID)
**if**  ( Viol == 0 and Speed == 0 ) {
        CompBonus = +5
} **else {**
        CompPenalty = Viol*2 + Speed*1
} **end** if
 
daysInact= daysSinceLastActivity(vehicleID)
**if** ( daysInact > 30 ) { 
        InactPenalty = floor(daysInact / 10) 
} **else** { 
        InactPenalty = 0 
} **end** if
NewRep = CurrentRep
NewRep = NewRep + ConsBonus - ConsPenalty
NewRep = NewRep + IntBonus  - IntPenalty
NewRep = NewRep + CompBonus - CompPenalty
NewRep = NewRep - InactPenalty
NewRep = clamp(NewRep, 0, 100)
setReputation(vehicleID, NewRep)
 
**return** NewRep
**End**

## 4. Simulation Results and Performance Evaluation

This section presents the formal security analysis, simulation environment and setup, and performance results of the HBV-IoT.

### 4.1. Security Analysis

The resilience of HBV-IoT is based on the combination of four mechanisms, namely: (i) cryptographic authentication and tamper evidence, (ii) interaction-based and Independent Transaction Validation at RSUs, (iii) reputation-weighted distributed consensus, and (iv) periodic state updates and malicious nodes. We describe the mitigation mechanisms for the primary attack types we consider in our threat model and provide a summary of the quantitative results.

1. Double-Spending Attack: In a double-spending attack, a compromised vehicle attempts to reuse the same credits or conflicting state in multiple transactions. HBV-IoT mitigates this in three ways:
  - Smart Contract 1 also ensures: at RSUs, temporal consistency and chain linkage between (PTID), and (TID) by rejecting non-monotonic or duplicate transaction sequences for the same vehicle.
  - The distributed consensus algorithm employs reputation-weighted voting, where reaching consensus in the presence of fraudulent transactions while including an arbitrary number of compromised RSUs requires a supermajority (≥70%) of weighted validator trust and is effectively impossible if most weight is honest.
  - In the central layer, when conflicting transactions are seen over time (e.g., two transactions spending the same credit), they are considered as strong evidence for the raised maliciousness score of the vehicle using DetectMaliciousNodes, and therefore, move it into ISOLATE/PENALIZE.
  Simulation results show that HBV-IoT detects double-spending at about 99% while maintaining false positives around 0.5%, and requires, on average, three suspicious events to classify a malicious vehicle, outperforming the VBCA and BDAV baselines.
2. Sybil Attack: In Sybil attacks, an adversary forges many vehicle identities to influence consensus or reputations. HBV-IoT prevents Sybil impact as follows.
  - Vehicle identities are bound to registration and long-term reputation; newly appearing identities start with moderate reputation and therefore low consensus weight, so they cannot immediately dominate RSU decisions.
  - Interaction-based transactions rely on cross-vehicle corroboration and physical constraints (relative position, speed, duration); Sybil identities that do not correspond to real vehicles cannot consistently produce plausible, mutually consistent interaction data.
  - The reputation update algorithm penalizes vehicles exhibiting repeated inconsistencies or anomalies, so clusters of Sybils with correlated misbehavior are gradually driven into low-reputation regions and eventually isolated.
  HBV-IoT attains Sybil attack detection rates above 96% with lower false positives and shorter detection times than the considered baselines, due to its combination of behavior-driven reputation and interaction validation.
3. A 51% Consensus Attack: To mitigate 51% consensus attack, the traffic device validators run a reputation-weighted voting process to prevent low-reputation nodes from controlling decisions. High-reputation RSUs influence the decision more than low-reputation ones. These distributed traffic device validators are selected across geographic network regions to prevent collusion within a single region. In simulations, HBV-IoT detects validator-level attacks (modeled as “51% consensus” scenarios) with rates above 92%, and requires fewer suspicious validation outcomes to isolate colluding nodes than conventional schemes.
4. Transaction Replay Attack: To prevent transaction replay attacks, each transaction contains a unique TID based on the content hash to prevent duplicates and also uses sequence numbers in the blockchain to prevent out-of-order insertion. Furthermore, the timestamp of each transaction is validated using *the Time_S() contract function,* and transactions older than 5 s are generally considered suspicious and are automatically rejected by the HBV-IoT model. It detects replay attacks at a rate near 99.8%, with almost zero false-positive rate and an average detection time of one suspicious event, significantly better than VBCA and BDAV.
5. Man-in-the-Middle Attack: To prevent a man-in-the-middle attack, the proposed HBV-IoT model utilizes ECDSA digital signatures using Ver_S() contract function to verify data integrity. Mutual authentication between vehicles and traffic devices prevents an attacker from intercepting vehicle-to-traffic device communication and modifying data. Furthermore, hash verification using the Mer_HV() contract function detects any modification to the transaction, and such trisections are liable to rejection. This prevents a man-in-the-middle attack in the HBV-IoT. As a result, HBV-IoT achieves MITM detection rates around 99%, with low false positives (~0.3%) and rapid classification of compromised nodes.

### 4.2. Simulation Environment

An urban HBV-IoT network is simulated by deploying vehicles over a 10 km × 10 km road grid, with network sizes ranging from 50 to 300 smart vehicles to evaluate scalability. The performance of HBV-IoT was evaluated using a custom discrete-event simulator that models smart vehicles, RSUs, and central servers, together with the proposed transaction formats, smart contracts, and consensus and reputation algorithms. Vehicles are equipped with on-board IoT sensors and generate 10–20 independent transactions per hour, while 5–10 interaction-based transactions per hour occur as vehicles come within a 100–300 m communication range of each other or roadside units/traffic devices. Roadside units and traffic devices are placed in a regular grid with approximately 500 m spacing, resulting in about 80–100 infrastructure nodes that act as layer-2 validators and edge-computing entities. The total simulation duration is set to 1000 real-time equivalent seconds, using wireless links with 5–25 Mbps of bandwidth per traffic device to emulate realistic urban connectivity conditions. Performance is assessed using latency (ms from generation to confirmation), throughput (transactions per second), average energy consumption per transaction (mJ/tx) at vehicles, malicious node-detection rate, validation success rate, and false-positive rate (honest nodes wrongly flagged as malicious) as the primary evaluation metrics. For a fair comparison, the DVRC, VBCA, and BDAV methods share the same mobility traces, transaction-generation processes, and communication parameters; only the validation, consensus, and storage logic differ. In HBV-IoT, we implement the four-layer hierarchical architecture with local buffering at vehicles, RSU-level independent and interaction-based transaction validation contracts, reputation-weighted distributed consensus at RSUs, and periodic malicious-node detection and reputation update at the central layer. Edge-level filtering removes invalid and low-quality transactions before they reach the global chain. VBCA follows the consortium vehicular blockchain model where RSUs jointly maintain a single-layer chain using a Raft-inspired leader-based consensus and basic transaction validation (signature, format, consistency) without interaction-based cross-checks or hierarchical offloading. In BDAV, we implemented a delay-aware vehicular data storage architecture in which vehicles send transactions through RSUs to edge/cloud storage nodes, which then participate in delay-aware consensus and query mechanisms. For DVRC, we implemented a Dynamic Vehicle Reputation Consensus scheme in which vehicle/validator reputation is embedded into block validation and leader selection on a single chain, without explicit edge-level filtering or hierarchical storage. Reputation values influence consensus weights, but all validated transactions are stored on the same global ledger. To assess variability and reliability, the simulation used the average of 30 runs to compare results with 95% confidence intervals computed using Student’s t-distribution for the HBV-IoT with state-of-the-art methods. The results of the proposed HBV-IoT model are compared with the DVRC, VBCA, and BDAV methods.

### 4.3. Thresholds Selection and Sensitivity

Table 2 shows a sensitivity analysis of the interaction time threshold, varying the time-difference threshold from 1 to 4 s. For the interaction time-difference threshold, we performed a sensitivity analysis by varying the threshold from 1 to 4 s. When the threshold is set to 1.0 s, the detector achieves an almost perfect malicious interaction detection rate (i.e., true positive rate, TPR) of 99.9%. Still, it incurs a false-positive rate (FPR) of about 1.9% on honest interactions. Relaxing the threshold to 1.5 s slightly reduces the detection rate to 98.7% while sharply reducing false positives to 0.4%, indicating that a small increase already improves robustness to timing jitter. At our chosen value of 2.0 s, the detector maintains a high detection rate of 91.7% and achieves 0% false positives, which we consider a practical operating point that balances security and reliability. For thresholds larger than 2.0 s, the detection rate drops rapidly (73.8% at 2.5 s, 49.9% at 3.0 s, and only 8.3% at 4.0 s) while the false-positive rate remains at 0%, showing that overly permissive thresholds significantly weaken the ability to flag replayed or stale interactions without yielding additional robustness benefits. The chosen 2 s threshold, therefore, represents a practical compromise, maintaining high interaction-validation success and strong replay resistance while being robust to realistic synchronization.

### 4.4. Performance Results

**Figure 3:**
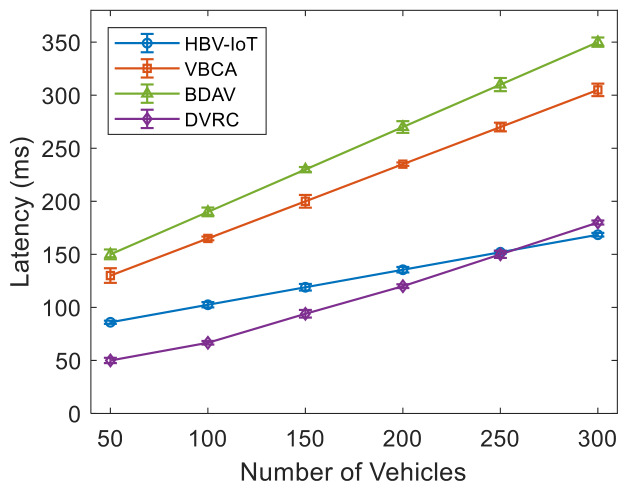
Latency performance of HBV-IoT.

Figure 3 shows the latency performance of the HBV-IoT model and state-of-the-art models for different numbers of vehicles. Each data point represents the mean over 30 independent runs, with error bars indicating 95% confidence intervals. It is observed that the proposed HBV-IoT consistently achieves lower end-to-end delay than both the VBCA and the BDAV across all vehicle counts. For example, for 50 vehicles, HBV-IoT takes 86 ms to write all transactions to the global blockchain, while VBCA takes 130 ms. The HBV-IoT achieves approximately 34% lower latency than VBCA. When the number of vehicles increases to 300, HBV-IoT takes 168.5 ms to write all transactions to the global blockchain, while VBCA takes 305 ms. HBV-IoT achieves 44.8% lower latency than VBCA. This shows that the latency gap widens as the network grows because the single-layer consensus and broadcast overhead in the VBCA-style design scale worse with the number of participants than the hierarchical HBV-IoT model. Similarly, at 50 vehicles, HBV-IoT achieves approximately 42.7% lower latency than the BDAV model. For 300 vehicles, HBV-IoT achieves approximately 51.8% lower latency than the BDAV model. Here, the hierarchical design benefits from local RSU-level consensus and batching, whereas BDAV storage architectures often push more operations to edge/cloud layers, adding extra hops and coordination delays. The latency of DVRC is lower up to 250 vehicles compared with HBV-IoT, but at 300 vehicles, it starts increasing sharply and is higher than HBV-IoT’s. The key points for lower latency for HBV-IoT are that vehicles only offload to nearby RSUs, which then aggregate and validate before sending concise batches to central servers, avoiding all-to-all communication among many nodes, lightweight, reputation-weighted consensus used at data acquisition layer-2, and invalid or low-quality transactions are discarded at RSUs before reaching blockchain layer-3, reducing load and queueing delay in central nodes relative to edge/cloud BDAV systems that store and process more raw data. HBV-IoT consistently achieves significantly lower end-to-end latency than VBCA, BDAV, and DVRC across all network sizes. The confidence intervals of HBV-IoT do not overlap with those of the baselines, confirming that the observed gains are statistically robust.

**Figure 4:**
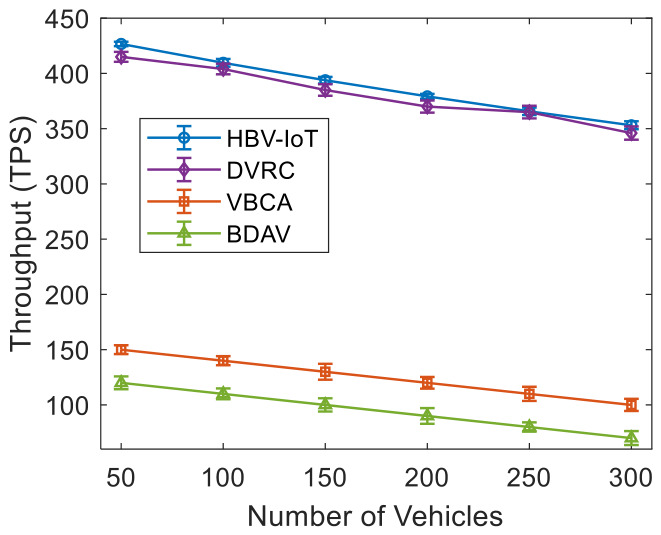
The throughput performance of HBV-IoT.

Figure 4 shows the throughput performance of the HBV-IoT model and its contemporary models for different numbers of vehicles. Figure 4 reports the throughput results averaged over 30 independent runs, with 95% confidence intervals. It is observed that HBV-IoT consistently achieves the highest throughput across all network sizes. Its confidence intervals remain clearly above those of DVRC, VBCA, and BDAV, indicating that the throughput gains are statistically robust. For example, for 50 vehicles, HBV-IoT processed 426 TPS. In contrast, DVRC achieves around 400 TPS, VBCA about 150 TPS, and BDAV about 120 TPS, so HBV-IoT provides roughly 2.8× and 3.6× higher throughput than VBCA and BDAV, respectively. When the number of vehicles increases to 300, HBV-IoT still processes about 353 TPS, while DVRC, VBCA, and BDAV reach roughly 335 TPS, 100 TPS, and 70 TPS, giving HBV-IoT about 3.5 times and 5.0 times higher throughput than VBCA and BDAV, respectively. The gap between TPS increases as DVRC and VBCA use single-layer consensus, and more communications are required as validators and vehicles grow. At the same time, HBV-IoT keeps most of the heavy work at RSUs with efficient batching and lightweight consensus. Similarly, at 50 and 300 vehicles, HBV-IoT provides 3.6 times and 5.0 times higher throughput, respectively, than BDAV. This is because VBCA storage/edge designs push more data toward edge/cloud storage and coordination layers, so each transaction incurs more I/O and synchronization, limiting system TPS compared to HBV-IoT’s localized validation and aggregation at RSUs. Furthermore, invalid or low-quality transactions are dropped early at RSUs, so the blockchain layer spends resources only on a smaller, high-quality subset, allowing HBV-IoT to maintain over 350 TPS even with 300 vehicles. At the same time, conventional baselines fall to 10–100 TPS.

**Figure 5:**
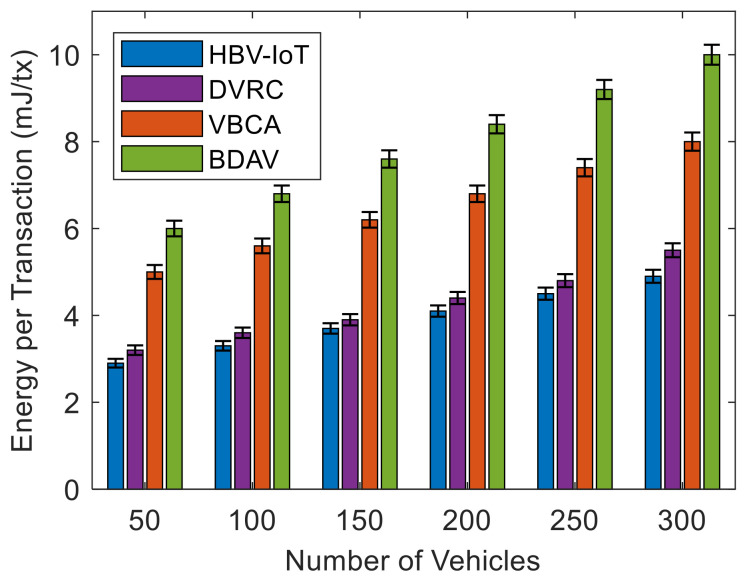
Energy usage analysis of HBV-IoT.

Figure 5 shows that HBV-IoT consumes the least energy per transaction across all network sizes from 50 to 300 vehicles. It is observed that HBV-IoT achieves the lowest energy per transaction across all network sizes, and its 95% confidence intervals remain narrow, indicating stable and repeatable energy savings over 30 runs. DVRC closely tracks HBV-IoT, with slightly higher means and overlapping intervals, indicating that it offers competitive but still inferior energy efficiency. In contrast, VBCA and the BDAV storage architecture exhibit substantially higher energy consumption with wider confidence intervals. It suggests that both experience heavier storage overhead and greater energy usage variability as the number of vehicles grows. For example, in HBV-IoT, energy rises moderately from 2.9 to 4.9 mJ/tx as network size increases from 50 to 300, reflecting modest extra work per transaction under higher load. The VBCA-like architecture rises from about 5.0 to 8.0 mJ/tx, staying roughly 45% higher than HBV-IoT across all sizes. This is because the VBCA single-layer consortium consensus requires more communication between validators and cryptographic operations per transaction. The BDAV increases from 6.0 to 10.0 mJ/tx, approximately 55% higher than HBV-IoT, driven by additional edge/cloud storage, replication, and coordination overhead per transaction. It is observed that for all numbers of vehicles that are up to 300, the result depicts that HBV-IoT’s hierarchical design with local buffering at vehicles, lightweight RSU-level consensus, and early filtering keeps per-transaction energy significantly lower (3–5 mJ per transaction) than conventional vehicular blockchain approaches, while still supporting higher throughput and lower latency.

**Figure 6:**
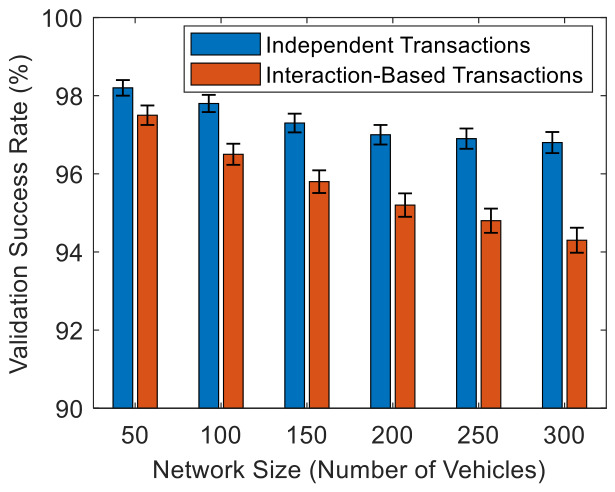
Transaction validation success rates.

Figure 6 shows the transaction validation success rates for independent and interaction-based transactions across different numbers of vehicles in the HBV-IoT. It is observed that both independent and interaction-based transactions maintain high success rates above 94% across all network sizes. Their 95% confidence intervals are narrow and indicate reliable, low-variance behavior across 30 runs. Independent transactions remain slightly more successful than interaction-based ones, particularly at larger network sizes, suggesting that they benefit more from the HBV-IoT validation pipeline. Independent transactions stay around 98.2% at 50 vehicles and slowly decrease to about 96.8% at 300 vehicles, indicating that almost all periodic vehicle status reports pass signature, timestamp, and plausibility even as the network scales. Interaction-based transactions start around 97.5% at 50 vehicles and decline to about 94.3% at 300 vehicles, slightly lower than independent ones because they must satisfy dual-vehicle consistency, physics checks, and synchronized timestamps, so more are rejected when there is any mismatch between the two vehicles’ data.

**Figure 7:**
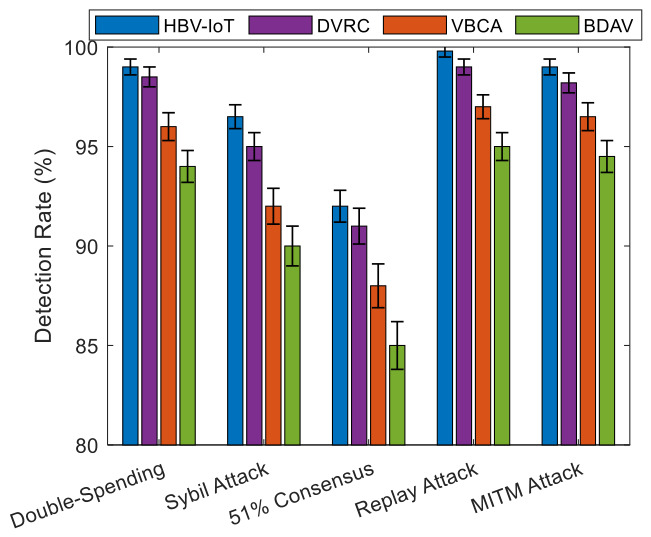
Malicious node-detection rate.

Figure 7 shows the malicious node-detection rate by attack types considered in the threat model for HBV-IoT. It is observed that the HBV-IoT detects all five attack types with accuracies of 92–99.8% and shows better detection than DVRC, VBCA, and BDAV blockchain storage/edge architectures for 50 malicious vehicles out of 300. For example, in the case of a double-spending attack, HBV-IoT improves detection by about 0.5%, 3%, and 5% compared with DVRC, VBCA, and BDAV, respectively. This indicates that almost all double-spend attempts in HBV-IoT are caught before confirmation. Similarly, for the Sybil attack, HBV-IoT achieves detection rates approximately 1% to 6.5% higher than those of DVRC, VBCA, and BDAV. This is due to HBV-IoT’s reputation-based and interaction-consistency checks, which track multiple identities’ behavior over time. For the five attacks considered for analysis, HBV-IoT improves detection rates by roughly 3–7% over VBCA and 5–10% over BDAV. This is because HBV-IoT evaluates every transaction using cryptographic checks, including signatures, hashes, IDs, and timestamps. And it uses interaction-based transactions so that another vehicle’s view immediately contradicts forged data and maintains dynamic reputation scores, so repeated small anomalies quickly accumulate into a strong signal for isolation. HBV-IoT consistently attains the highest detection rates with narrow intervals across all attack types, indicating both accurate and stable identification of malicious vehicles. DVRC remains close to HBV-IoT, whereas VBCA and the IoV storage architecture show lower means and wider confidence intervals, revealing a higher likelihood of missed attacks and more variable behavior.

**Figure 8:**
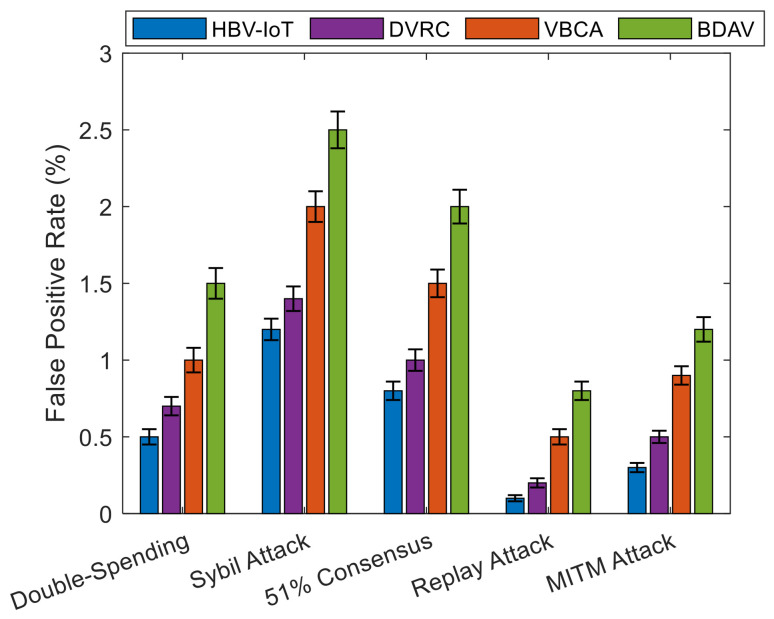
False-positive rate by attack type.

Figure 8 shows the false-positive rate for the different types of attacks considered in the threat model for HBV-IoT. It is observed that in HBV-IoT, the false-positive rate stays below about 1.5%, whereas in DVRC, VBCA, and VBCA, it can rise up to 2 to 2.5%. This is because HBV-IoT cross-checks data from multiple vehicles, and the malicious-node detection contract accumulates inconsistency rates, temporal–spatial anomalies, and violation patterns, then computes a maliciousness score. Only when this score passes a threshold does the node move from “MONITOR” to “ISOLATE” or “PENALIZE.” This smoothing over multiple transactions reduces the chance that noise, sensor error, or a transient communication glitch will trigger a false alarm. The results show that HBV-IoT is not only better at catching real attacks, but also safer for honest vehicles, which are much less likely to be unfairly isolated or penalized.

**Figure 9:**
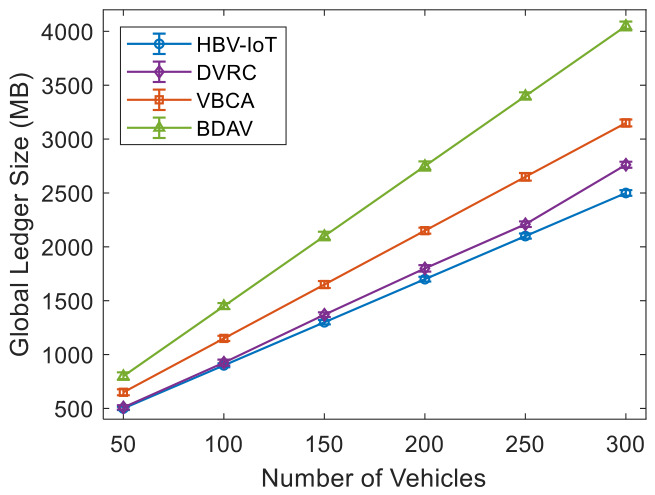
Ledger size at central server.

**Figure 10:**
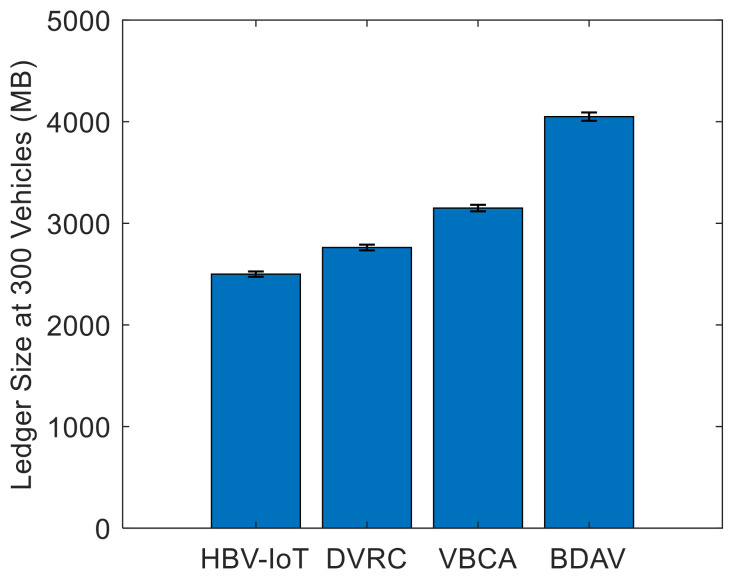
Ledger size at 300 vehicles.

Figure 9 shows the global blockchain size at the central server for different numbers of vehicles. It is observed that as the number of vehicles increases, the ledger size increases more slowly than DVRC, BVCA, and DBCA. HBV-IoT shows the smallest ledger growth across all network sizes. Its 95% confidence intervals are relatively narrow. This indicates reduced storage overhead and stable behavior over 30 runs. DVRC remains consistently above HBV-IoT in ledger size. However, it is still lighter than VBCA and the BDAB IoV storage architecture. These two baselines have clearly larger mean ledger sizes. Their confidence intervals are also wider, suggesting more variability in storage growth. At 300 vehicles, as shown in Figure 10, the intervals of HBV-IoT do not overlap with those of the baselines. This lack of overlap supports the conclusion that the hierarchical design’s storage savings are statistically robust. For example, there are 300 vehicles as shown in Figure 10. The ledger size for HBV-IoT is approximately 2.5 GB, while it is 3.15 GB and 4.05 GB for VBCA and DBAV, respectively. This means HBV-IoT reduces the central ledger size by about 20% compared with VBCA and by about 38% compared with DBCA, as shown in Table 3. This is because in HBV-IoT, aggregation and pruning transactions at RSUs before sending them to the blockchain layer, rejecting invalid transactions at RSUs instead of storing everything in the central blockchain, and interaction-based deduplication, where only one canonical copy of corroborated interaction data is kept centrally, rather than both vehicles’ versions.

### 4.5. Discussion

In relation to the existing literature, HBV-IoT can be seen as integrating and extending several previously related works that primarily focus on improving consensus latency and trust management within a single-layer vehicular blockchain. In contrast, HBV-IoT provides local buffering at the vehicle level, RSU-level independent and interaction-based transaction validation, reputation-weighted distributed consensus, and periodic malicious-node detection at the central layer, all within a single framework. RSU-level independent and interaction-based transaction validation smart contracts allow early edge-level filtering of vehicular transactions. Only plausibly correct and cross-verified transactions are forwarded to the global blockchain. This edge-level filtering reduces central processing overhead, ledger size, and improves data quality for downstream applications. This localized validation enables RSUs to spot local anomalies or conflicting interaction records quickly. Consequently, this limits the impact of malicious vehicles on the HBV-IoT system. A reputation-weighted distributed consensus algorithm strengthens the HBV-IoT control plane by giving greater influence to validators (RSUs) with a proven history of honest and reliable behavior. This approach reduces the chance that a small group of newly created or intermittently malicious nodes can bias block decisions. Instead of treating all validators equally, consensus outcomes are determined by votes, which naturally mitigates Sybil attacks. This approach discourages small-scale malicious behavior because any detected deviation lowers reputation, thereby reducing future voting power. At the same time, the algorithm reduces coordination overhead by selecting high-reputation validators as leaders, improving throughput and latency without sacrificing security.

Periodic malicious-node detection at the central layer: RSU-level checks by analyzing transaction histories, consensus logs, and the reputation of all vehicles to identify slow or suspicious nodes that local validators fail to detect in isolation. The central analysis periodically renews the reputation and the operational condition of every vehicle and RSU (e.g., MONITOR, ISOLATE, PENALIZE). These updates are fed into RSU-level validation and reputation-weighted consensus, forming a closed loop in which observed misbehavior is instantly turned into less influence and stronger filtering. This helps keep false positives low. The proposed smart contacts and algorithms make HBV-IoT a more scalable, energy-efficient, and secure foundation for real-time traffic monitoring, e-challan, insurance, and vehicle registration in vehicular IoT networks.

The results show two notable effects. A 2 s interaction-based validation window provides almost the same replay-attack detection as a 1 s window, while eliminating false positives, making the system more robust to clock and network imperfections. In addition, DVRC has slightly lower latency than HBV-IoT only at a small scale (up to about 250 vehicles), but its performance collapses as the network grows. In contrast, HBV-IoT maintains stable latency by decoupling heavy reputation handling from per-transaction processing. The results report mean values from 30 independent runs, with 95% confidence intervals computed using Student’s t-distribution. Across all metrics, HBV-IoT not only achieves the best absolute performance but also exhibits relatively narrow intervals, indicating low variability and stable behavior under different network sizes and attack types. For most points, the confidence intervals of HBV-IoT do not overlap with those of DVRC, VBCA, and the BDAV, supporting the conclusion that the observed latency, throughput, detection, and storage gains are statistically significant rather than due to random fluctuations.

## 5. Conclusions and Future Work

In this paper, we propose HBV-IoT, a hierarchical blockchain architecture for vehicular IoT networks that enables smart vehicles to join the global blockchain network and securely exchange information with each other. Independent and interaction-based transactions enabling multi-source validation and fraud detection are presented. Smart contracts for automated validation, consensus, and dispute resolution mechanisms are presented. Algorithms for achieving consensus and detecting malicious nodes in vehicular networks are presented. The security analysis of the proposed HBV-IoT model against various attack types is presented. The simulation results from 30 independent runs, reported with 95% confidence intervals, show that the HBV-IoT model incurs latencies of 86–168 ms to process all transactions across network scales. The throughput of the proposed BHV-IoT is about three times that of contemporary models, and its hierarchical design keeps per-transaction energy significantly lower than conventional vehicular blockchain Proof-of-Work approaches, while still supporting higher throughput and lower latency. The proposed model achieves a detection rate of about 94% for malicious nodes with less than 2% false positives. This model can be used by individual vehicle owners, insurance companies, and government entities to develop better ideas, solutions, and services for vehicular traffic control and safety. Apart from this, we have discussed some use cases where it can be used in the automobile industry and traffic control to provide a better solution and service to deal with problems related to these industries, and have described how this model can help to come up with a better solution than existing technologies if implemented properly.

## Figures and Tables

**Table 1:** Analytical analysis of state-of-the-art methods and HBV-IoT.

Methods	Ledger Layering	RSU Role	Interaction-Based Validation	Consensus Type	Attack Coverage	Storage/Growth Management	Scalability with Density
DVRC [12]	Single chain with a reputation score	Validators and reputation updaters	Indirect, via accumulated behavior	Reputation-aware BFT	No storage or replay	No	Degrades at high density
VBCA [10]	Single chain at RSUs	Validators/miners	Not explicit	Lightweight consortium/Raft-like consensus	Basic blockchain resilience	No	Degrades at high density
BDAV [11]	Single or dual chain	Data collectors and local storage nodes	Not explicit	Delay-aware consensus	Data integrity	Optimized placement	Degrades at high density
BTMR [14]	Single-layer	Collect trust evidence, maintain trust metrics	Indirect via multi-source trust scores	Lightweight consensus	Targets false information, malicious	Optimized trust storage, but it still grows with interactions	Degrades at high density
BECA [18]	Dual blockchain	Verifiers	Not explicit	Consortium-style consensus	Identity and auth-phase attacks	Sensitive vs public info separated across chains	Degrades at high density
Proposed HBV-IoT	Four-layer hierarchy (vehicle, RSU, central, application)	Independent and interaction validation, consensus	Explicit dual-signed interaction transactions	Reputation-weighted RSU consensus with periodic central malicious-node detection.	Combined replay, Sybil, double-spend, 51%, MITM via edge filtering and reputation loop	Local buffering/filtering, selective forwarding, and reduced duplicate interactions on the chain.	Maintains more stable latency and throughput at high density via hierarchy and edge filtering

**Table 2:** Sensitivity of the interaction time-difference threshold.

Time-Difference		
Threshold (s)	TPR (%)	FPR (%)
1.0	99.9	1.92
1.5	98.7	0.4
2.0	91.7	0.0
2.5	73.8	0.0
3.0	49.9	0.0
3.5	26.7	0.0
4.0	8.3	0.0

**Table 3:** Ledger size and space efficiency.

Vehicles	HBV-IoT	Ledger Size in MB			% HBV-IoT Space Efficiency		
		DVRC	VBCA	BDAV	DVRC	VBCA	BDAV
50	500	510	650	800	1.96	23.07	37.50
100	900	925	1150	1450	2.70	21.73	37.93
150	1300	1370	1650	2100	5.10	21.21	38.09
200	1700	1800	2150	2750	5.55	20.93	38.18
250	2100	2210	2650	3400	4.97	20.75	38.23
300	2500	2762	3150	4050	9.48	20.63	38.27

## Data Availability

All data generated or analyzed during this study are included in this published article.
